# Development and validation of a multiplex TaqMan real-time quantitative PCR assay for the simultaneous detection of six major bacterial respiratory pathogens in goats

**DOI:** 10.3389/fvets.2026.1925484

**Published:** 2026-09-17

**Authors:** Mingzhe Fu, Yi Liu, Zeyang Zhang, Weimin Li, Qi Zhang, Xingang Xu

**Affiliations:** 1 College of Veterinary Medicine, Northwest A&F University, Yangling, Shaanxi, China; 2 Datong District Animal Husbandry Comprehensive Service Center, Daqing, Heilongjiang, China

**Keywords:** bacterial and mycoplasmal pathogens, co-infection, goat respiratory disease, molecular diagnosis, multiplex TaqMan qPCR, small ruminants

## Abstract

**Background:**

Respiratory disease caused by bacterial and mycoplasmal pathogens is an important threat to goat health, and mixed infections complicate etiological diagnosis. Rapid multiplex detection is therefore valuable for clinical investigation and surveillance.

**Methods:**

Two triplex TaqMan real-time quantitative PCR (qPCR) panels were established for the simultaneous detection of six major caprine respiratory pathogens. Panel I targeted *Mycoplasma ovipneumoniae* (Mo), *Mycoplasma mycoides* subsp. *capri* (Mmc), and *Mycoplasma capricolum* subsp. *capripneumoniae* (Mccp), whereas Panel II targeted *Pasteurella multocida* (Pm), *Mannheimia haemolytica* (Mh), and *Streptococcus equi* subsp. *zooepidemicus* (Sz). Analytical specificity was assessed using target plasmids and nucleic acids from target and non-target pathogens, whereas analytical sensitivity and reproducibility were evaluated using recombinant plasmid standards. Clinical applicability was assessed separately using 200 field-collected goat nasal swab samples.

**Results:**

No amplification was observed for the tested non-target organisms. The limits of detection were 1.73 × 10^2^ copies/μL for Mo, 2.01 × 10^3^ copies/μL for Mmc, 3.63 × 10^2^ copies/μL for Mccp, 1.03 × 10^2^ copies/μL for Pm, 1.59 × 10^2^ copies/μL for Mh, and 5.88 × 10^2^ copies/μL for Sz. Intra-assay CVs ranged from 0.55 to 2.76%, and inter-assay CVs ranged from 0.38 to 3.10%. Multiplex qPCR detected at least one target in 117 of 200 clinical samples (58.5%), including 47 samples with two or more targets (23.5%).

**Conclusion:**

The multiplex qPCR assay developed in this study provides a rapid and specific tool for the simultaneous detection of six major respiratory pathogens in goats, with potential utility for clinical diagnosis and epidemiological surveillance.

## 1 Introduction

Respiratory diseases are among the most important constraints on goat production worldwide, causing mortality, impaired weight gain, reduced milk production, and substantial treatment and prevention costs (1–3). Their etiology is complex and commonly involves bacterial, mycoplasmal, and viral agents, often in mixed infections. Chinese epidemiological data demonstrate both the importance and geographical variability of this problem: in Hainan, *Mycoplasma ovipneumoniae* seroprevalence was 31.7%, with PCR positivity of 7.3% in nasal swabs and 26.1% in pneumonic lung tissues (4); in Anhui, 27.5% seropositivity was reported during a goat-pneumonia investigation (5); and in Sichuan, *Pasteurella multocida* showed an overall isolation rate of 9.71%, with location-specific PCR detection ranging from 0 to 23.25% (6). Although quantified economic estimates from China remain limited, a prospective Kenyan study projected annual contagious caprine pleuropneumonia losses of approximately EUR 1,713 in a typical 100-goat herd, illustrating the herd-level consequences of delayed detection and control (7). These findings underscore the need for rapid etiological diagnosis of caprine respiratory disease.

The clinically relevant pathogen spectrum includes six bacterial and mycoplasmal agents with complementary pathogenic roles. *Mycoplasma ovipneumoniae* can impair respiratory defense and predispose animals to secondary bacterial pneumonia (5, 8–12). *Mycoplasma capricolum subsp. capripneumoniae* causes contagious caprine pleuropneumonia and belongs to the closely related *M. mycoides cluster*, whereas *Mycoplasma mycoides subsp. capri* can produce pneumonia, mastitis, arthritis, and systemic mycoplasmosis (13–22). *Pasteurella multocida* and *Mannheimia haemolytica* are major causes of pneumonic pasteurellosis and bronchopneumonia, while *Streptococcus equi subsp. zooepidemicus* can act as an opportunistic respiratory pathogen in small ruminants (23–30). Because these agents can coexist in affected airways, detection of only one pathogen may not adequately characterize the etiological complexity of a clinical case.

Accurate etiological diagnosis remains difficult because culture, biochemical identification, and serology can be slow or insufficiently sensitive, particularly for fastidious *Mycoplasma* species, while single-target PCR requires separate reactions, greater sample volume, and additional reagent and handling time (19, 23, 31). Molecular methods are available for individual targets, and published small-ruminant multiplex assays have progressed from duplex conventional PCR to three-target qPCR and four-target PCR or RT-qPCR formats (12, 20, 25, 32–35). However, these assays detect only limited subsets of respiratory pathogens and do not provide coordinated coverage of all six agents described above. Consequently, suspected mixed infections still require several separate tests, increasing sample consumption, turnaround time, and cost and potentially leaving co-infections incompletely characterized. A clear diagnostic gap therefore remains for rapid and coordinated molecular detection of these six clinically relevant pathogens in goats.

Therefore, this study aimed to develop and evaluate a coordinated multiplex TaqMan real-time qPCR approach for detecting these six major bacterial and mycoplasmal respiratory pathogens in goats.

## 2 Materials and methods

### 2.1 Bacterial strains, primer and probe design

Bacterial strains, including *M. ovipneumoniae*, *M. mycoides* subsp. *capri*, *M. capricolum* subsp. *capripneumoniae*, *P. multocida*, *S. equi* subsp. *zooepidemicus*, and *M. haemolytica*, were stored at −80 °C in our laboratory. At least 15 genome sequences of each pathogen were downloaded from NCBI and analyzed using MEGA software (Version 11.0). Multiple sequence alignment was performed using the ClustalW algorithm implemented in MEGA version 11.0 with default parameters. Candidate conserved regions were selected based on high nucleotide sequence conservation among the available sequences of each target pathogen. Regions containing nucleotide variations at or near the 3′ ends of candidate primers or within the probe-binding sites were excluded. After comparison, the Hsp70 gene of *M. ovipneumoniae*, the LppA gene of *M. mycoides* subsp. *capri*, the MOLPALP gene of *M. capricolum* subsp. *capripneumoniae*, the Kmt1 gene of *P. multocida*, the EF-Tu gene of *S. equi* subsp. *zooepidemicus*, and the 16S rRNA gene of *M. haemolytica* were found to be the most conserved gene sequences and selected as target genes. Primers and probes were designed with Beacon Designer 8 software (Premier Biosoft, Palo Alto, CA, USA) targeting the most conserved region within each gene. The melting temperatures and GC contents of all primers and probes, as well as potential probe hairpin structures, were evaluated using OLIGO Primer Analysis Software version 7 (Molecular Biology Insights, Inc., Cascade, CO, USA). The target specificity of the candidate primers and probes was further assessed using NCBI BLAST. Fluorophore and quencher labels were excluded from the thermodynamic calculations. Table 1 displays the sequences of the primers and probes developed in this study, along with their fluorophore and quencher assignments; detailed thermodynamic parameters are provided in Supplementary Table S2A. TsingKe Biological Technology Co., Ltd. (Beijing, China) synthesized the primers and probes.

**Table 1:** Primers and probes used in the two triplex qPCR panels.

Panel	Target	Oligo	Gene	Sequence (5′–3′)	Dye/Quencher	Amplicon (bp)	Final conc. (μM)
I	Mo	Mo-F	Hsp70	CAATCAAACCGAAGTTACAAT	–	116	0.30
		Mo-R		GAAGACCACGAGGAGCAG	–		0.30
		Mo-P		CGATTCCTGAAAGATTAAAGCGACC	HEX/BHQ1		0.25
	Mmc	Mmc-F	LppA	TACCAACGATCAAAAACAACCAA	–	108	0.30
		Mmc-R		TCTGGTTGTTTTGGATTATGAGA	–		0.30
		Mmc-P		TATTTTCTGGCTTACCGGGGCTTG	FAM/BHQ1		0.30
	Mccp	Mccp-F	MOLPALP	CCAGCATTTGTTCCACTATT	–	142	0.10
		Mccp-R		GTCGTGTAAATAAAAAAGAAAATG	–		0.10
		Mccp-P		ACAGACTAAGACAGTTTGCTGCTA	CY5/BHQ2		0.05
II	Pm	Pm-F	Kmt1	CTCAGCTTATTGTTATTTG	–	133	0.15
		Pm-R		ACACGATAAATAAATTAAACC	–		0.15
		Pm-P		CGGTTTATATTTCCTTGTCAGTCTG	HEX/BHQ1		0.25
	Mh	Mh-F	16S rRNA	ACGGTAGCACATAGGAGT	–	126	0.15
		Mh-R		CCTAAGACATTACGCGGTA	–		0.15
		Mh-P		CCGTTTCCAGTAGTTATCCCCCT	FAM/BHQ1		0.30
	Sz	Sz-F	EF-Tu	GGTGAAGTATACATCCTTACTAA	–	142	0.10
		Sz-R		CAGGCATAACCATTTCTGTA	–		0.10
		Sz-P		CACTCCATTCTTCAACAACTACCGTC	CY5/BHQ2		0.05

### 2.2 Nucleic acid extraction

For bacterial strains and clinical specimens, nasal swabs were first homogenized in phosphate-buffered saline. To enhance cell lysis prior to extraction, both bacterial cultures and homogenized clinical samples were subjected to three freeze–thaw cycles, each consisting of freezing at −80 °C for 10 min followed by thawing at 25 °C for 5 min. Total nucleic acids were subsequently extracted from all samples using a commercial DNA extraction kit (Cat: B0916A, Tiangen Biotech Co., Ltd., Beijing, China) according to the manufacturer’s instructions. The concentration and A260/A280 purity ratio of the extracted DNA were determined with a NanoDrop spectrophotometer (Thermo Fisher Scientific, Waltham, MA, USA). The extracted DNA was stored at −80 °C until further use.

### 2.3 Preparation of standard control plasmids

The target gene fragments for each pathogen were amplified by conventional PCR using the designed primers. The PCR products were verified by 2% agarose gel electrophoresis, purified using a PCR purification kit (Cat: B1224A, Tiangen Biotech Co., Ltd., Beijing, China), and cloned into the pLB-T vector (Cat: VT217, Tiangen Biotech Co., Ltd., Beijing, China) according to the manufacturer’s instructions. The recombinant plasmids were transformed into *Escherichia coli* DH5α competent cells. Positive clones were selected and confirmed by Sanger sequencing (TsingKe Biological Technology Co., Ltd., Beijing, China). Recombinant plasmids were purified using an Endotoxin-Free Plasmid Mini/Midi Kit (Spin Column; Tiangen Biotech Co., Ltd., Beijing, China; catalog no. DP118-02) according to the manufacturer’s instructions. Plasmid concentrations and purity were determined using a NanoDrop 2000 spectrophotometer (Thermo Fisher Scientific, Wilmington, DE, USA). Three replicate spectrophotometric readings were obtained for each plasmid. The mean concentrations, A260/A280 ratios, and A260/A230 ratios are provided in Supplementary Table S2B. Plasmid copy numbers were calculated using the formula:

$$\text{Copies}/\mathrm{\mu L}=\frac{6.02\times 10^{23}\times \text{plasmid concentration}\;(\mathrm{ng}/\mathrm{\mu L})\times 10^{-9}}{\text{plasmid length}\;(\mathrm{bp})\times 660}$$

In the copy-number calculation, plasmid concentration is expressed in ng/μL, plasmid length in base pairs (bp), 660 g/mol is the mean molecular mass of one base pair of double-stranded DNA, and 6.022 × 10^23^ molecules/mol is Avogadro’s constant. The resulting value is reported as plasmid copies/μL.

The final stock concentrations were 4.23 × 10^10^ copies/μL (Mo), 7.39 × 10^10^ copies/μL (Mmc), 3.77 × 10^10^ copies/μL (Mccp), 1.04 × 10^11^ copies/μL (Pm), 7.84 × 10^10^ copies/μL (Sz), and 5.01 × 10^10^ copies/μL (Mh). For multiplex standard curve analysis, the recombinant plasmids corresponding to each panel were normalized to an identical copy number and mixed in equal volumes. Panel I consisted of Mo, Mmc, and Mccp plasmids, and Panel II consisted of Pm, Mh, and Sz plasmids. The mixed plasmid standards were then subjected to tenfold serial dilution to generate a series of standards for each multiplex panel. These standards were used to construct standard curves by plotting Cq values against the logarithm of plasmid copy numbers. Each dilution was tested in triplicate.

### 2.4 Optimization of the multiplex qPCR assay

To establish a robust detection system, the assay was divided into two separate triplex panels: Panel I (Mo, Mmc, Mccp) and Panel II (Pm, Mh, Sz). Primer concentrations were first screened separately for each target in uniplex reactions. Equal final concentrations of the forward and reverse primers were evaluated at 0.10, 0.20, 0.30, and 0.40 μM, while the plasmid input, probe concentration, total reaction volume, and cycling conditions were kept constant. The 20 μL total reaction volume was retained in accordance with the recommended format of the probe qPCR master mix and was not treated as an optimized variable. Primer selection was based on the lowest concentration that produced a near-minimal and stable Cq value together with a typical sigmoidal amplification profile and adequate fluorescence. The uniplex primer-concentration screening curves are provided in Supplementary Figure S4.

Following the uniplex screening, primer inputs were further balanced during assembly of the corresponding triplex panel. An intermediate concentration of 0.15 μM was evaluated for Pm and Mh between the 0.10 and 0.20 μM screening levels. Probe concentrations were initially screened at 0.05, 0.10, 0.20, and 0.30 μM and were subsequently fine-adjusted within the best-performing concentration intervals when necessary. For each panel, probe screening was performed individually in a triplex amplification background containing the three equal-copy plasmid templates and all three primer pairs; one target-specific probe was evaluated at a time, while the other two probes were omitted. The selected probes were subsequently combined and verified in the complete triplex reactions. Optimization was considered acceptable when the selected oligonucleotide inputs produced stable Cq values, typical sigmoidal curves, adequate channel-specific fluorescence, no quantifiable NTC amplification within 40 cycles, and no target dropout after assembly of the complete triplex reaction. When increasing an oligonucleotide concentration produced no meaningful improvement, the lower concentration was selected. The probe-concentration screening range and final selected concentrations are summarized in Supplementary Table S3, and the final primer and probe concentrations are reported in Table 1. The multiplex qPCR was conducted on a TL988 Real-Time Fluorescence Quantitative PCR System (Xi’an Tianlong Science and Technology Co., Ltd., Xi’an, China) using 2 × Probe Master Mix (Cat: E091-01A, Vazyme, Nanjing, China), 2 μL of DNA template, and the final primer and probe concentrations shown in Table 1. The thermal cycling program was initial denaturation at 95 °C for 2 min, followed by 40 cycles of denaturation at 95 °C for 10 s and annealing/extension at 57 °C for 20 s. The annealing/extension temperature was selected by considering the predicted melting temperatures of the primers and probes, the recommended working conditions of the master mix, and the simultaneous amplification performance of all three targets in each panel during preliminary optimization. A temperature of 57 °C provided stable amplification of all six targets and was selected for subsequent experiments. Fluorescence signals were acquired during extension, and NTCs were included in every run. Each clinical sample was analyzed in two reactions, one for each panel; thus, the assay was a two-panel triplex workflow rather than a single-tube six-plex reaction.

### 2.5 Analytical specificity, sensitivity, and reproducibility

Analytical specificity was evaluated using nucleic acid preparations from the six target pathogens and six non-target pathogens: *Mycoplasma bovis*, *Escherichia coli*, bovine viral diarrhea virus (BVDV), *Mycoplasma arginini*, *Trueperella pyogenes*, and bovine herpesvirus 1 (BoHV-1; infectious bovine rhinotracheitis virus, IBRV). The standard plasmids of the six target pathogens served as positive controls, and nuclease-free water served as the negative control. To evaluate amplification competition, the three target plasmids in each triplex panel were normalized to the same high-copy concentration and tested individually or as an equal-copy three-target mixture under identical multiplex qPCR conditions. The per-target plasmid input in each mixed reaction was kept the same as that used in the corresponding single-template reaction. The limit of detection (LOD) was determined using tenfold serial dilutions of mixed standard plasmids, from 10^6^ to 10^0^ copies/μL. Each dilution was tested in three replicates. The LOD was defined as the lowest plasmid concentration at which all three replicates produced positive amplification with Cq values below 40. The repeatability of the multiplex qPCR assay was evaluated using standard plasmids at concentrations of 10^6^, 10^5^, and 10^4^ copies/μL. Each reaction was performed in triplicate. Intra-assay repeatability was assessed by testing three replicates of each dilution under the same conditions. Inter-assay repeatability was assessed by repeating the assay on three separate days. The coefficient of variation (CV) of the Cq values was calculated to evaluate repeatability. Standard-curve slopes, coefficients of determination (*R*^2^), and amplification efficiencies for all six targets are reported in Figure 1. For conventional PCR sensitivity visualization, amplification products were separated on a 1% agarose gel at 170 V for 25 min using a D2000 DNA Marker (catalog no. BL102A).

**Figure 1:**
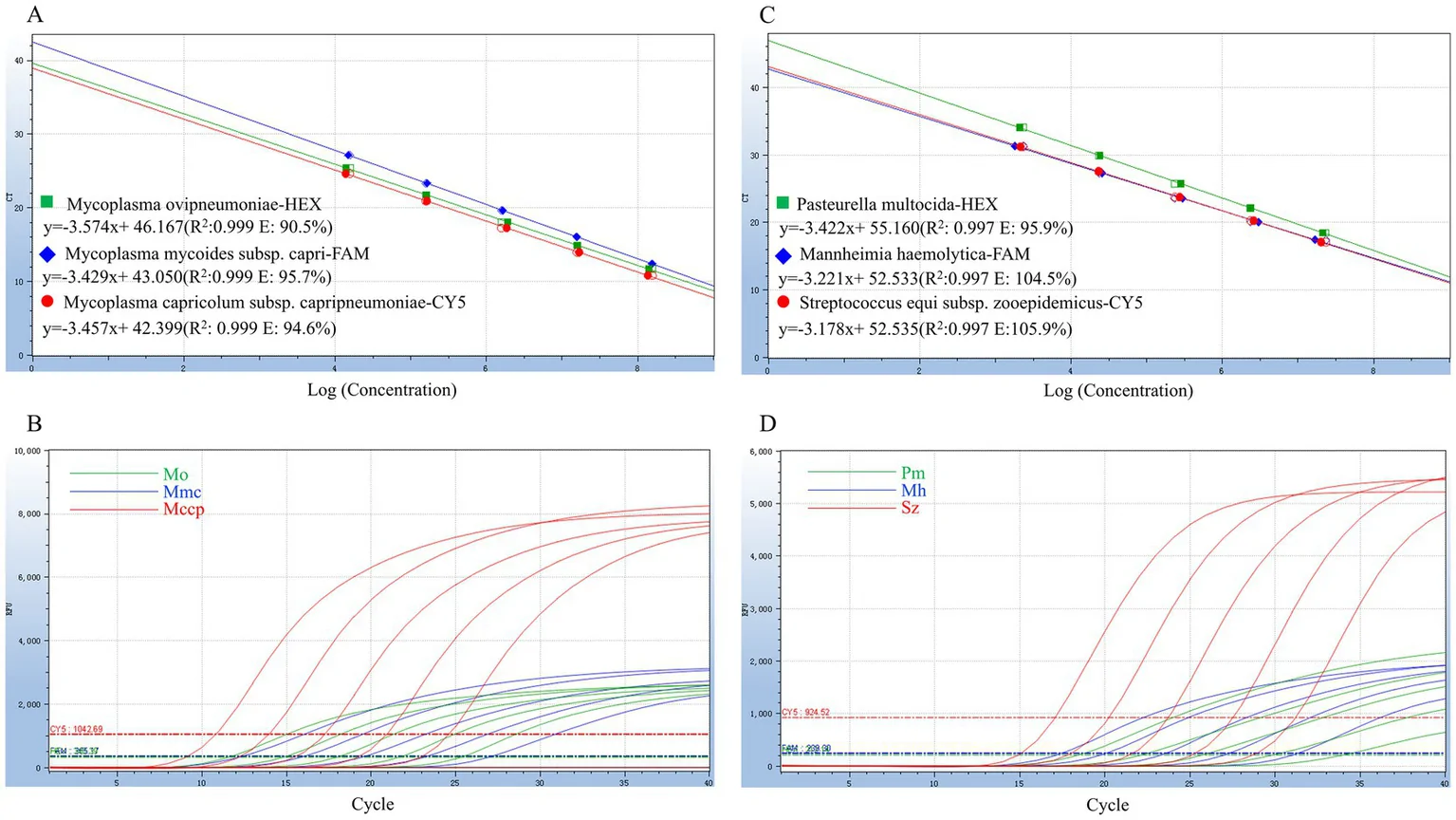
Standard curves and amplification plots of the multiplex qPCR assay for the six target pathogens. Ten-fold serial dilutions of mixed standard plasmids (10^7^–10^3^ copies/μL for Panel I and 10^8^–10^4^ copies/μL for Panel II) were tested. **(A)** Standard curves for Panel I (Mo, Mmc, Mccp) showing linear relationship between Cq values and log copy numbers, with slope and *R*^2^ values indicated for each target. **(B)** Amplification plots for Panel I. **(C)** Standard curves for Panel II (Pm, Mh, Sz) showing linear relationship between Cq values and log copy numbers, with slope and *R*^2^ values indicated for each target. **(D)** Amplification plots for Panel II.

### 2.6 Clinical sample testing

A total of 200 goat nasal swab samples were collected from goat farms in Shaanxi and Heilongjiang provinces, China, between 2024 and 2025. The sampled goats were naturally occurring clinical cases or suspected cases of respiratory disease. Animals were selected when they showed one or more respiratory signs, including nasal discharge, coughing, sneezing, dyspnea or increased respiratory effort, fever, or abnormal respiratory sounds. Nasal swabs were collected aseptically by trained personnel, placed into sterile tubes containing phosphate-buffered saline, transported to the laboratory on ice, and stored at −80 °C until nucleic acid extraction.

All samples were collected with the permission of the farm owners. No experimental infection, drug treatment, surgery, or other invasive animal procedure was performed. The study used only non-invasive nasal swab samples collected from naturally occurring field cases for pathogen detection.

All 200 clinical goat nasal swab samples were tested using the established multiplex qPCR assay. In parallel, all samples were tested by routine PCR using the same primer pairs. The positive rate was calculated as the number of positive samples divided by the total number of clinical samples tested (*n* = 200). Samples positive for one target pathogen were classified as single infections, whereas samples positive for two or more target pathogens were classified as co-infections.

### 2.7 Statistical analysis

The standard curves were generated by plotting the logarithm of plasmid copy numbers against the corresponding Cq values. Linear regression analysis was performed to calculate the slope, coefficient of determination (*R*^2^), and amplification efficiency (*E*) for each target. Cq values were automatically calculated using the manufacturer-supplied analysis software of the TL988 Real-Time Fluorescence Quantitative PCR System. The baseline and fluorescence thresholds were automatically determined by the instrument software and applied consistently to all samples. A sample was considered positive when it produced a typical sigmoidal amplification curve that crossed the automatically determined fluorescence threshold before cycle 40 (Cq < 40); samples that did not cross the threshold within 40 cycles or did not exhibit a typical sigmoidal curve were considered negative. Clinical detection results are presented as numbers and percentages. Because multiplex qPCR and routine PCR were applied to the same 200 clinical samples, pathogen-specific positive rates were compared using McNemar’s chi-square test; the two-sided exact McNemar test was used because the numbers of discordant pairs were small. Agreement between the two methods was evaluated using Cohen’s kappa coefficient. Co-infection patterns were summarized descriptively as numbers and percentages. All statistical analyses were performed using GraphPad Prism version 9.0 (GraphPad Software, San Diego, CA, USA). All tests were two-sided, and *p* < 0.05 was considered statistically significant.

## 3 Results

### 3.1 Establishment of the multiplex qPCR assay

During uniplex primer-concentration screening, Mo, Mmc, Pm, and Mh showed earlier and comparable amplification at 0.30 and 0.40 μM, whereas increasing the concentration to 0.40 μM produced no meaningful additional improvement. Accordingly, 0.30 μM was selected as the provisional concentration for these four targets. In contrast, Mccp and Sz showed similar amplification profiles across the tested range of 0.10–0.40 μM, and the lowest concentration of 0.10 μM was therefore selected (Supplementary Figure S4). During subsequent triplex balancing, the primer concentrations for Pm and Mh were further refined to 0.15 μM, while Mo and Mmc were retained at 0.30 μM and Mccp and Sz at 0.10 μM. Following initial probe screening at 0.05, 0.10, 0.20, and 0.30 μM and target-specific fine adjustment within this range, the final probe concentrations were 0.25, 0.30, and 0.05 μM for Mo, Mmc, and Mccp, respectively, and 0.25, 0.30, and 0.05 μM for Pm, Mh, and Sz, respectively (Supplementary Table S3). At the final concentrations shown in Table 1, both complete triplex panels generated typical target-specific sigmoidal amplification curves, with no target dropout in the equal-copy mixtures and no quantifiable amplification in the NTCs within 40 cycles (Supplementary Figures S1, S2).

### 3.2 Standard curves and analytical specificity

Tenfold serial dilutions of the mixed standard plasmids were then used to generate standard curves. For Panel I (Mo, Mmc, Mccp), concentrations ranged from 10^7^ to 10^3^ copies/μL; for Panel II (Pm, Mh, Sz), concentrations ranged from 10^8^ to 10^4^ copies/μL. Thus, both panels were evaluated across five consecutive tenfold concentrations. All six targets showed strong linear relationships between log plasmid copy number and Cq, with *R*^2^ values exceeding 0.99 and amplification efficiencies of 90–110% (Figure 1). Analytical specificity was evaluated against the six target pathogens and six non-target pathogens: *Mycoplasma bovis*, *Escherichia coli*, BVDV, *Mycoplasma arginini*, *Trueperella pyogenes*, and BoHV-1/IBRV. No cross-amplification was observed for any tested non-target pathogen or the negative control (Figure 2 and Supplementary Figure S3). In the high-copy plasmid interference experiments, all three targets in each panel retained clear sigmoidal amplification as an equal-copy mixture, with no target dropout under the tested conditions (Supplementary Figures S1, S2).

**Figure 2:**
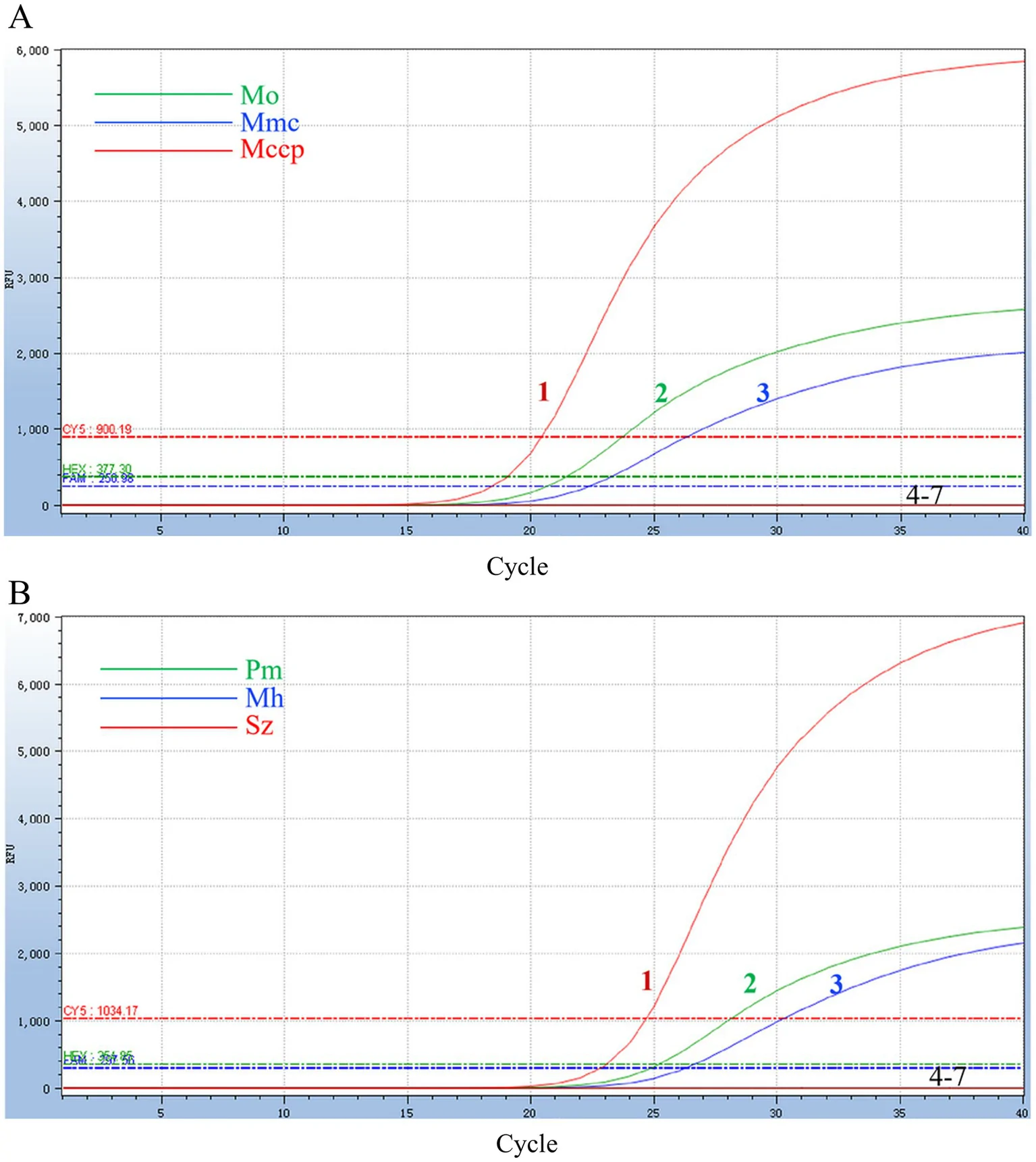
Analytical specificity evaluation of the multiplex qPCR assay. **(A)** Specificity testing for Panel I targets (1. Mo, 2. Mmc, 3. Mccp) against the initially tested non-target organisms (4. *Mycoplasma bovis*, 5. *Escherichia coli*, 6. BVDV) and negative control (7. ddH_2_O). **(B)** Specificity testing for Panel II targets (1. Pm, 2. Mh, 3. Sz) against the same non-target organisms and negative control. Only the target pathogens produced amplification signals; no cross-amplification was detected for non-target organisms or the negative control.

### 3.3 Analytical sensitivity

Under the optimized reaction conditions, ten-fold serial dilutions of the recombinant plasmid standards ranging from 10^6^ to 10^0^ copies/μL were used to evaluate the analytical sensitivity of the multiplex TaqMan real-time qPCR assays (Figure 3). The limits of detection (LOD) for the six target pathogens were determined as follows: 1.73 × 10^2^ copies/μL for Mo, 2.01 × 10^3^ copies/μL for Mmc, 3.63 × 10^2^ copies/μL for Mccp, 1.03 × 10^2^ copies/μL for Pm, 1.59 × 10^2^ copies/μL for Mh, and 5.88 × 10^2^ copies/μL for Sz.

**Figure 3:**
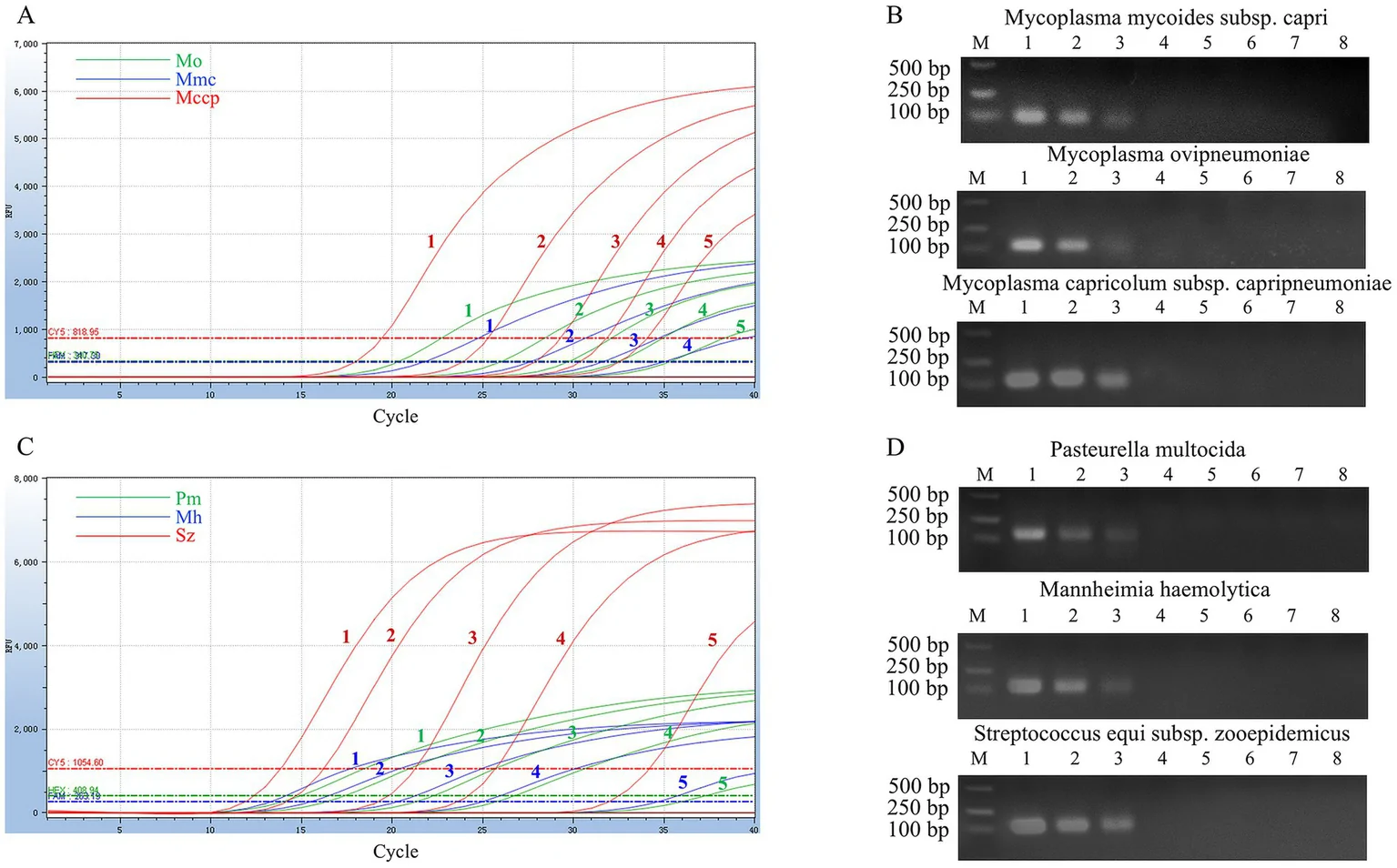
Analytical sensitivity of the multiplex qPCR assay. Ten-fold serial dilutions of mixed standard plasmids (10^6^–10^0^ copies/μL) were tested. **(A)** Multiplex qPCR amplification curves for Panel I targets (Mo, Mmc, Mccp). **(B)** Conventional PCR detection of Panel I targets. **(C)** Multiplex qPCR amplification curves for Panel II targets (Pm, Mh, Sz). **(D)** Conventional PCR detection of Panel II targets. M: D2000 DNA Marker; lanes 1–7: 10^6^, 10^5^, 10^4^, 10^3^, 10^2^, 10^1^, and 10^0^ copies/μL, respectively; lane 8: ddH_2_O negative control. The visible D2000 DNA Marker bands are labeled at 500, 250, and 100 bp in panels B and D.

### 3.4 Reproducibility

The reproducibility of the multiplex qPCR assay was evaluated using standard plasmids at 10^6^, 10^5^, and 10^4^ copies/μL. As shown in Table 2, intra-assay CV values ranged from 0.55 to 2.76%, and inter-assay CV values ranged from 0.38 to 3.10%, demonstrating low within-run and between-run variability across the tested concentrations.

**Table 2:** The repeatability tests of the multiplex qPCR.

Panel	Templates	Concentrations (copies/μL)	Inter-assay		Intra-assay	
			Cq values (mean ± SD)	CV%	Cq values (mean ± SD)	CV%
I	*M. ovipneumoniae*	10^4^	28.35 ± 0.42	2.12	28.15 ± 0.47	1.67
		10^5^	23.74 ± 0.58	2.45	24.50 ± 0.28	1.14
		10^6^	19.41 ± 0.41	1.48	19.87 ± 0.11	0.55
	*M. mycoides* subsp. *capri*	10^4^	30.64 ± 0.31	1.02	30.05 ± 0.83	2.76
		10^5^	26.47 ± 0.82	3.10	27.23 ± 0.71	2.61
		10^6^	20.11 ± 0.17	0.85	21.60 ± 0.21	0.97
	*M. capricolum* subsp. *capripneumoniae*	10^4^	27.63 ± 0.64	2.32	28.77 ± 0.79	2.75
		10^5^	23.42 ± 0.09	0.38	25.10 ± 0.49	1.95
		10^6^	19.90 ± 0.12	0.60	20.75 ± 0.16	0.77
II	*P. multocida*	10^4^	28.92 ± 0.44	1.52	31.02 ± 0.85	2.74
		10^5^	25.80 ± 0.67	2.60	24.18 ± 0.24	0.99
		10^6^	22.45 ± 0.31	1.38	22.25 ± 0.27	1.21
	*M. haemolytica*	10^4^	27.33 ± 0.82	3.00	25.88 ± 0.62	2.39
		10^5^	24.67 ± 0.22	0.89	22.71 ± 0.36	1.59
		10^6^	20.10 ± 0.15	0.75	20.34 ± 0.14	0.69
	*S. equi* subsp. *zooepidemicus*	10^4^	29.40 ± 0.76	2.59	29.30 ± 0.68	2.32
		10^5^	26.18 ± 0.53	2.02	26.44 ± 0.55	2.08
		10^6^	21.05 ± 0.19	0.90	23.92 ± 0.33	1.38

### 3.5 Clinical sample testing

The established multiplex qPCR assay was used to detect six target respiratory pathogens in 200 goat nasal swab samples. Overall, 117 samples were positive for at least one target pathogen, corresponding to an overall detection rate of 58.5% (117/200). Co-infections involving two or more target pathogens were detected in 47 samples, accounting for 23.5% of all clinical samples. Among the 117 multiplex qPCR-positive samples, 70 samples were positive for a single pathogen and 47 samples were positive for two or more pathogens. For individual pathogens, the multiplex qPCR assay detected Mo in 49 samples (24.5%), Mmc in 43 samples (21.5%), Mccp in 26 samples (13.0%), Pm in 26 samples (13.0%), Mh in 21 samples (10.5%), and Sz in 26 samples (13.0%). Routine PCR detected Mo in 37 samples (18.5%), Mmc in 40 samples (20.0%), Mccp in 25 samples (12.5%), Pm in 16 samples (8.0%), Mh in 15 samples (7.5%), and Sz in 22 samples (11.0%). The three co-infection patterns observed by multiplex qPCR were Mo + Pm in 26 samples (13.0%), Mo + Mmc + Mh in 15 samples (7.5%), and Mo + Mccp+Mh + Sz in 6 samples (3.0%). These three patterns accounted for all 47 co-infection cases detected by multiplex qPCR and are shown in Figure 4B. The Mo + Mccp+Mh + Sz pattern represented a four-pathogen co-infection; no five- or six-pathogen combinations were observed. The corresponding detection rates by routine PCR were 8.0% (16/200), 5.5% (11/200), and 2.0% (4/200), respectively.

**Figure 4:**
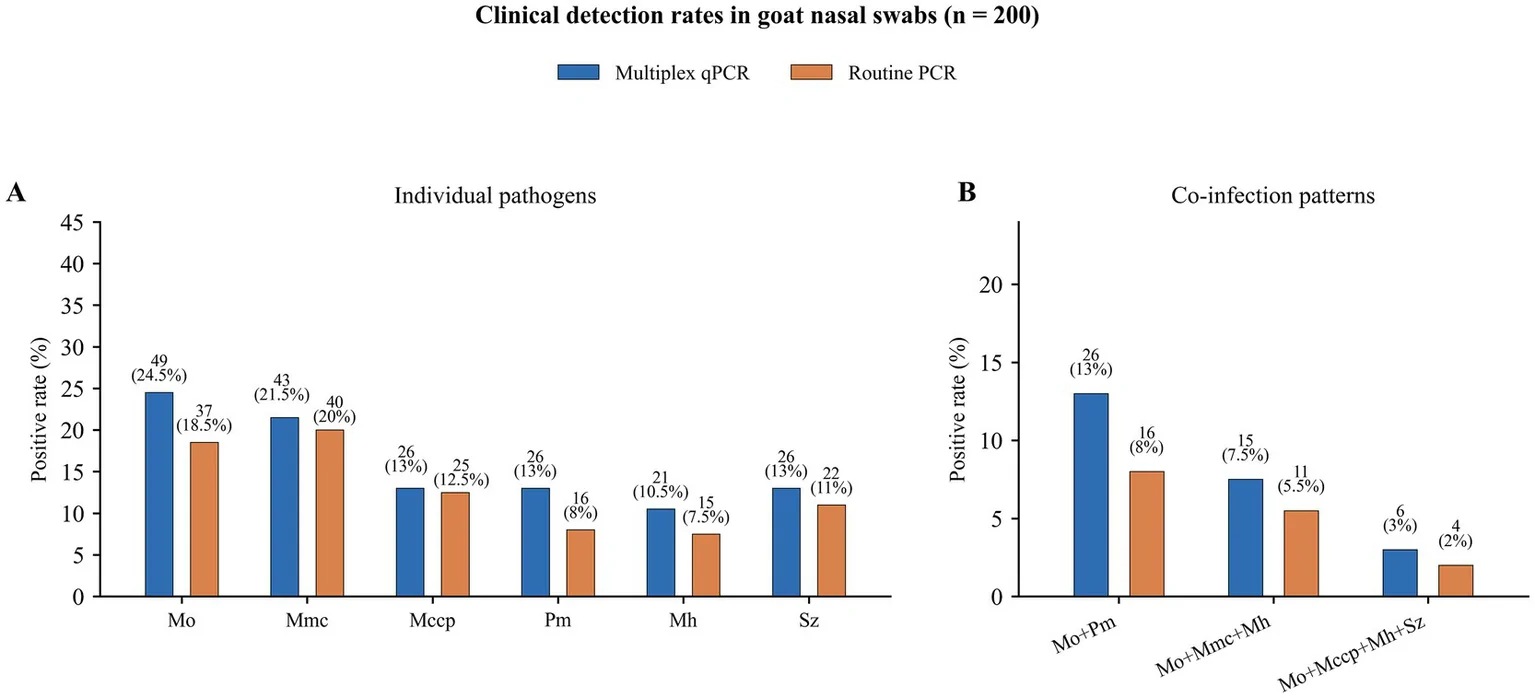
Clinical detection of six respiratory pathogens in 200 goat nasal swab samples using multiplex qPCR and routine PCR **(A)** Positive rates of individual pathogens detected by multiplex qPCR and routine PCR **(B)** Detection rates of co-infection patterns identified in clinical samples. Numbers above bars indicate the number of positive samples and the corresponding positive rate. Positive rates were calculated as the number of positive samples divided by the total number of clinical samples tested (*n* = 200).

All samples that tested positive by routine PCR were also positive by multiplex qPCR. Based on the paired results, overall agreement between the two methods ranged from 94.0 to 99.5%, and Cohen’s kappa coefficients ranged from 0.736 to 0.978. Multiplex qPCR additionally detected 12 Mo-positive, 3 Mmc-positive, 1 Mccp-positive, 10 Pm-positive, 6 Mh-positive, and 4 Sz-positive samples that were negative by routine PCR. Two-sided exact McNemar tests identified significant differences in positive rates for Mo (*p* < 0.001), Pm (*p* = 0.002), and Mh (*p* = 0.031), whereas the differences for Mmc (*p* = 0.250), Mccp (*p* = 1.000), and Sz (*p* = 0.125) were not statistically significant. The complete paired 2 × 2 contingency data and agreement statistics are provided in Supplementary Table S1.

## 4 Discussion

Respiratory disease remains a major constraint on goat production because the detected agents can act as primary pathogens, opportunists, or components of polymicrobial disease. Mo may impair mucociliary defense and facilitate secondary bacterial invasion, Mccp and Mmc can cause severe pleuropneumonia or systemic mycoplasmosis, and Pm, Mh, and Sz may exploit damaged or stressed airways. Consequently, identifying a single organism does not always explain the full clinical syndrome, while simultaneous detection can provide a more informative basis for differential diagnosis, herd-level control, and targeted confirmatory testing (1–3, 10, 13, 21–28). In the present study, the two-panel multiplex TaqMan qPCR assay was designed to detect these six biologically complementary respiratory targets within one coordinated workflow.

None of the six targets is entirely without a molecular detection method, but the scope and analytical validation of available assays differ. For Mo, single-target and differential real-time PCR assays have been reported (12, 36). Mccp can be identified by species-specific PCR and has also been included with *P. multocida* in a four-target small-ruminant RT-qPCR assay (19–20). *P. multocida* and Mh have been detected by duplex or four-plex conventional PCR, and molecular assays are also available for *P. multocida* species identification and Mh capsular characterization (25, 29–30, 33, 35). Real-time PCR has been described for detection and differentiation of *S. equi* subspecies, including Sz (23). Mmc has been identified molecularly in clinical and experimental studies, but directly comparable analytical validation data for a caprine respiratory multiplex qPCR remain limited (21–22). Taken together, these studies show that each target can be detected individually or within a limited multiplex format. The principal advance of the present assay is therefore not the first molecular detection of any individual organism, but the coordinated coverage of all six clinically relevant bacterial and mycoplasmal targets. Existing small-ruminant respiratory multiplex assays generally cover only two to four targets, whereas the present workflow integrates all six targets into one diagnostic strategy (12, 20, 25, 32–35).

Analytically, all six targets showed good standard-curve linearity, with *R*^2^ values greater than 0.99 and amplification efficiencies within the commonly accepted range for real-time PCR assays (37). The LODs ranged from 1.03 × 10^2^ to 2.01 × 10^3^ copies/μL, and five targets were detected within the 10^2^ copies/μL range. The Mo LOD of 1.73 × 10^2^ copies/μL was in the same order of magnitude as a published real-time PCR LOD of 10^2^ copies per reaction, although another singleplex qPCR using genomic DNA detected 22 copies per reaction (36). For Pm and Mccp, a four-target small-ruminant RT-qPCR reported LODs of 13–163 copies per reaction (20). A recently reported ovine four-plex conventional PCR targeting Mo, Mh, and Pm achieved 10^2^–10^3^ copies/μL, which brackets the corresponding values of 1.03 × 10^2^ to 5.88 × 10^2^ copies/μL obtained here (35). Directly comparable qPCR LOD data for Mmc and Sz in the same specimen and multiplex format remain scarce. Overall, the five targets in the 10^2^ copies/μL range were broadly comparable in order of magnitude with published multi-target systems.

The principal sensitivity exception was Mmc, which showed a higher LOD of 2.01 × 10^3^ copies/μL. This difference may reflect local target-sequence context or secondary structure affecting primer–probe accessibility, together with competition for shared reagents near the detection limit. The high-copy mixture experiment showed no target dropout but did not exclude small Cq shifts in low-copy mixtures; this explanation therefore remains plausible rather than confirmed. Clinically, any reduction in analytical sensitivity would be most relevant to specimens with pathogen loads close to the detection limit, particularly low-burden or carrier-state Mmc samples. In the present cohort, however, every routine PCR-positive sample was also positive by multiplex qPCR, indicating that the measured analytical difference did not produce false-negative results relative to routine PCR in these 200 samples. When clinical suspicion remains high despite a negative multiplex result, repeat extraction, single-target qPCR, testing of a lower-respiratory-tract specimen when clinically appropriate, or confirmatory culture or sequencing should still be considered.

The intra-assay CVs of 0.55–2.76% and inter-assay CVs of 0.38–3.10% indicate stable Cq measurement across the tested concentrations. These ranges overlap the 2–4% variability reported for a four-target small-ruminant RT-qPCR, except at the lowest Mccp concentration (20), and are narrower than the upper limits reported for a canine respiratory four-plex panel (intra-run <6% and inter-run <5%) (38). They are, however, slightly broader than the <2% CVs reported for an eight-target bovine respiratory multiplex qPCR (39). Such differences do not necessarily indicate inferior assay performance: CV estimates depend on template concentration, number of replicates and independent runs, nucleic-acid type, fluorescence channel, platform, and whether variation is calculated from Cq values or transformed copy numbers. The present values are therefore within the range of comparable veterinary multiplex assays, although reproducibility should still be confirmed between laboratories.

The clinical detection rates also require population-specific interpretation. The Mo rate of 24.5% in the present symptomatic or suspected cohort was close to the 27.5% seropositivity reported in an Anhui pneumonia investigation (5), but exceeded the 7.3% nasal-swab PCR rate in a broader Hainan survey (4). The Pm rate of 13.0% was modestly higher than the 9.71% overall isolation rate reported in Sichuan but fell within that study’s location-specific PCR range of 0–23.25% (6). In contrast, the Mh rate of 10.5% was lower than the 60% detection rate among respiratory cases in a Lao PDR case–control study (3). These differences are plausible because the studies varied in geography, season, herd structure, disease severity, specimen type, and methodology (serology, culture, conventional PCR, or qPCR). The present samples were enriched for goats with respiratory signs but came from only two provinces, so the observed percentages should not be generalized as national prevalence estimates. Comparisons for Mmc, Mccp, and Sz are additionally constrained by limited directly comparable caprine nasal-swab surveys.

The co-detection patterns provide biologically plausible, but not causal, evidence of polymicrobial respiratory disease. Mo + Pm was the most frequent combination (13.0%), consistent with a model in which mycoplasmal colonization or epithelial injury facilitates expansion of an opportunistic Pasteurellaceae member. The Mo + Mmc + Mh pattern (7.5%) and Mo + Mccp + Mh + Sz pattern (3.0%) suggest that mycoplasmal and bacterial agents can coexist in clinically affected airways and may contribute different inflammatory or tissue-damaging mechanisms. However, qPCR detects target nucleic acid rather than organism viability, temporal sequence, or lesion attribution. These combinations should therefore guide confirmatory culture, sequencing, pathological assessment, and treatment decisions rather than be interpreted as proof that every detected organism contributed equally to disease. The absence of five- or six-target combinations may reflect the limited cohort and geography rather than a biological exclusion of such patterns.

From a practical perspective, the two-panel design provides a compromise between diagnostic breadth and assay robustness. It reduces six singleplex reactions to two wells per sample, uses only three fluorescence channels per panel, and avoids post-amplification gel handling. Compared with a single six-plex reaction, the split-panel format is easier to optimize and less vulnerable to extreme template imbalance; compared with singleplex testing, it reduces reagent, sample, and plastic consumption as well as hands-on processing. These features improve throughput and make the workflow compatible with conventional real-time PCR instruments. The trade-off is that two reactions are still required and the assay does not provide the complete one-tube simplicity of a higher-order multiplex. Similar panel-based approaches have been used in canine and bovine respiratory disease systems, whereas the present target combination is tailored to caprine bacterial and mycoplasmal respiratory disease (38–44).

This study has limitations in analytical, clinical, and interpretive validation. Analytically, sensitivity was established using purified plasmids, which may overestimate performance in inhibitor-rich clinical matrices; a dedicated matrix-interference experiment was not performed, and no internal amplification control was included. Although *M. bovis* and *M. arginini* were tested as non-target mycoplasmas, the specificity panel did not include additional members of the *M. mycoides* cluster or other mycoplasmas more closely related to Mo or Mmc, so exclusivity against the closest phylogenetic relatives remains incompletely established. In addition, the equal-copy high-template interference experiment did not reproduce the unequal, low-copy mixtures expected in field specimens. Clinically, evaluation was restricted to nasal swabs from only two provinces and primarily involved clinically affected or suspected goats; the observed detection rates may therefore be influenced by selection bias and should not be interpreted as national prevalence estimates. Routine PCR was not an independent reference standard, and discordant results were not confirmed by culture or sequencing. Interpretively, qPCR co-detection cannot distinguish active infection from carriage or determine the relative pathological contribution of each target. Future work should therefore include broader cross-reactivity testing, low-copy unequal-template competition and matrix-interference experiments, an internal control, blinded multicenter validation in several specimen types, and confirmation using culture, sequencing, and pathological findings where applicable.

In conclusion, the multiplex TaqMan qPCR assay developed in this study provides a rapid and practical molecular tool for the simultaneous detection of six major respiratory pathogens in goats. Its favorable analytical performance under the tested conditions, combined with its practical two-panel design, supports its potential application in clinical diagnosis and epidemiological surveillance. Further validation with larger, geographically diverse sample cohorts is needed to fully establish its utility in routine veterinary diagnostic settings.

## Data Availability

The raw data supporting the conclusions of this article will be made available by the authors, without undue reservation.

## Glossary

BVDV: bovine viral diarrhea virus
BoHV-1: bovine herpesvirus 1
IBRV: infectious bovine rhinotracheitis virus
CCPP: contagious caprine pleuropneumonia
Cq: quantification cycle
CV: coefficient of variation
EF-Tu: elongation factor Tu
Hsp70: heat shock protein 70
LOD: limit of detection
LppA: lipoprotein A gene
Mccp: *Mycoplasma capricolum* subsp. *capripneumoniae*
Mh: *Mannheimia haemolytica*
Mmc: *Mycoplasma mycoides* subsp. *capri*
Mo: *Mycoplasma ovipneumoniae*
NTC: no-template control
Pm: *Pasteurella multocida*
PPRV: peste des petits ruminants virus
qPCR: real-time quantitative polymerase chain reaction
*R* ^2^: coefficient of determination
RT-qPCR: reverse transcription quantitative polymerase chain reaction
Sz: *Streptococcus equi* subsp. *zooepidemicus*

## References

[1] Brogden KA Lehmkuhl HD Cutlip RC. *Pasteurella haemolytica* complicated respiratory infections in sheep and goats. Vet Res. (1998) 29:233–54. doi: 10.1016/S0928-4249(98)80026-6, 9689740

[2] Fox KA MacGlover CAW Blecha KA Stenglein MD. Assessing shared respiratory pathogens between domestic (*Ovis aries*) and bighorn (*Ovis canadensis*) sheep; methods for multiplex PCR, amplicon sequencing, and bioinformatics to characterize respiratory flora. PLoS One. (2023) 18:e0293062. doi: 10.1371/journal.pone.0293062, 37856492 PMC10586700

[3] Jayasekara PP Jenkins C Kirkland PD Gerber PF Olmo L Xaikhue T . *Mycoplasma ovipneumoniae* identified as the main aetiological agent of respiratory disease in goats from a case-control study in Savannakhet province of Lao PDR. Vet Microbiol. (2025) 301:110353. doi: 10.1016/j.vetmic.2024.110353, 39721247

[4] Rong G Zhao JM Hou GY Zhou HL. Seroprevalence and molecular detection of *Mycoplasma ovipneumoniae* in goats in tropical China. Trop Anim Health Prod. (2014) 46:1491–5. doi: 10.1007/s11250-014-0645-y, 25099398

[5] Chen J Wang S Dong D Zhang Z Huang Y Zhang Y. Isolation and characterization of *Mycoplasma ovipneumoniae* infecting goats with pneumonia in Anhui Province, China. Life (Basel). (2024) 14:218. doi: 10.3390/life14020218, 38398727 PMC10890177

[6] Li S Gu Q Li B Abi K Yang F. High prevalence of virulence genes and multi-drug resistance in *Pasteurella multocida* from goats in Sichuan, China. Vet J. (2025) 311:106344. doi: 10.1016/j.tvjl.2025.106344 40174797

[7] Renault V Hambe HA Van Vlaenderen G Timmermans E Mohamed AM Ethgen O . Economic impact of contagious caprine pleuropneumonia and cost-benefit analysis of the vaccination programmes based on a one-year continuous monitoring of flocks in the arid and semi-arid lands of Kenya. Transbound Emerg Dis. (2019) 66:2523–36. doi: 10.1111/tbed.13317, 31376342

[8] Pavone S Crotti S D'Avino N Gobbi P Scoccia E Pesca C . The role of *Mycoplasma ovipneumoniae* and *Mycoplasma arginini* in the respiratory mycoplasmosis of sheep and goats in Italy: correlation of molecular data with histopathological features. Res Vet Sci. (2023) 163:104983. doi: 10.1016/j.rvsc.2023.104983, 37639802

[9] Udhayavel S Sukumar K Senthilkumar K Srinivasan P Elango A. Isolation and molecular characterization of *Mycoplasma ovipneumoniae* associated with respiratory infection in sheep and goats in South India. Braz J Microbiol. (2025) 56:2087–95. doi: 10.1007/s42770-025-01663-4, 40287597 PMC12350858

[10] Dassanayake RP Shanthalingam S Herndon CN Subramaniam R Lawrence PK Bavananthasivam J . *Mycoplasma ovipneumoniae* can predispose bighorn sheep to fatal *Mannheimia haemolytica* pneumonia. Vet Microbiol. (2010) 145:354–9. doi: 10.1016/j.vetmic.2010.04.011, 20466492

[11] Weyand LK Felts BL Cassirer EF Jenks JA Walsh DP Besser TE. Fatal interactions: pneumonia in bighorn lambs following experimental exposure to carriers of *Mycoplasma ovipneumoniae*. J Clin Microbiol. (2025) 63:e01328-24. doi: 10.1128/jcm.01328-24, 39835822 PMC11837494

[12] Noll LW Highland MA Hamill VA Tsui WNT Porter EP Lu N . Development of a real-time PCR assay for detection and differentiation of *Mycoplasma ovipneumoniae* and a novel respiratory-associated *Mycoplasma* species in domestic sheep and goats. Transbound Emerg Dis. (2022) 69:e1460–8. doi: 10.1111/tbed.14477, 35166453 PMC9790229

[13] Nicholas R Churchward C. Contagious caprine pleuropneumonia: new aspects of an old disease. Transbound Emerg Dis. (2012) 59:189–96. doi: 10.1111/j.1865-1682.2011.01262.x, 21951488

[14] Soayfane Z Houshaymi B Nicholas RAJ. *Mycoplasma capricolum* subsp. *capripneumoniae*, the cause of contagious caprine pleuropneumonia, comprises two distinct biochemical groups. Open Vet J. (2018) 8:393–400. doi: 10.4314/ovj.v8i4.7, 30538929 PMC6243207

[15] Rahman MH Alam MS Ali MZ Haque MN Akther S Ahmed S. First report of contagious caprine pleuropneumonia (CCPP) in Bangladeshi goats: seroprevalence, risk factors and molecular detection from lung samples. Heliyon. (2024) 10:e40507. doi: 10.1016/j.heliyon.2024.e40507, 39717600 PMC11665336

[16] Zegeye A Jemberu WT Fentie T Mekonnen SA Molla W. Spatial distribution and clustering of contagious caprine pleuropneumonia in goats of Amhara region, Ethiopia. Heliyon. (2024) 10:e38180. doi: 10.1016/j.heliyon.2024.e38180, 39386806 PMC11462329

[17] Ahmad F Khan FA Khan H Saeed M. Determination of anti-*Mycoplasma capricolum* subsp. *capripneumoniae* antibodies for the sero-prevalence of contagious caprine pleuropneumonia in North-Western Pakistan. Pak J Zool. (2025) 57:531–8. doi: 10.17582/journal.pjz/20230205100201

[18] Khan H Khan FA Sadique U Ahmad I Ahmad F Rahman HU . Development and evaluation of inactivated whole-cell contagious caprine pleuropneumonia vaccine against *Mycoplasma capricolum* subsp. *capripneumoniae* Pakistan strain. Pak J Zool. (2026) 58:379–87. doi: 10.17582/journal.pjz/20240114191217

[19] Woubit S Lorenzon S Peyraud A Manso-Silvan L Thiaucourt F. A specific PCR for the identification of *Mycoplasma capricolum* subsp. *capripneumoniae*, the causative agent of contagious caprine pleuropneumonia (CCPP). Vet Microbiol. (2004) 104:125–32. doi: 10.1016/j.vetmic.2004.08.006 15530747

[20] Settypalli TBK Lamien CE Spergser J Lelenta M Wade A Gelaye E . One-step multiplex RT-qPCR assay for the detection of peste des petits ruminants virus, capripoxvirus, *Pasteurella multocida* and *Mycoplasma capricolum* subspecies (ssp.) capripneumoniae. PLoS One. (2016) 11:e0153688. doi: 10.1371/journal.pone.0153688, 27123588 PMC4849753

[21] Wagner H Heller M Fawzy A Schnee C Nesseler A Kaim U . *Mycoplasma mycoides* subspecies capri, an uncommon mastitis and respiratory pathogen isolated in a German flock of goats. Vet Microbiol. (2024) 290:109996. doi: 10.1016/j.vetmic.2024.109996, 38295490

[22] Di Provvido A. Scacchia M. Varasano V. Churchward C. De Caro C. Al-Momani W., et al. Experimental infection of goats with an unusual strain of *Mycoplasma mycoides* subsp. capri isolated in Jordan. J Comp Pathol (2009) 141:121–126. doi: 10.1016/j.jcpa.2008.12.007 19446837

[23] Baverud V Johansson SK Aspan A. Real-time PCR for detection and differentiation of *Streptococcus equi* subsp. *equi* and *Streptococcus equi* subsp. *zooepidemicus*. Vet Microbiol. (2007) 124:219–29. doi: 10.1016/j.vetmic.2007.04.020, 17531409

[24] Steward KF Robinson C Holden MTG Harris SR Ros AF Chacon-Perez G . Diversity of *Streptococcus equi* subsp. *zooepidemicus* strains isolated from the Spanish sheep and goat population and the identification, function and prevalence of a novel arbutin utilisation system. Vet Microbiol. (2017) 207:231–8. doi: 10.1016/j.vetmic.2017.06.020 28757029

[25] Sahay S Natesan K Prajapati A Kalleshmurthy T Shome BR Rahman H . Prevalence and antibiotic susceptibility of *Mannheimia haemolytica* and *Pasteurella multocida* isolated from ovine respiratory infection: a study from Karnataka, southern India. Vet World. (2020) 13:1947–54. doi: 10.14202/vetworld.2020.1947-1954 PMC7566252 33132610

[26] Alemu S Belachew Y Tefera T. Isolation and molecular detection of *Mannheimia haemolytica* and *Pasteurella multocida* from clinically pneumonic pasteurellosis cases of Bonga sheep breed and their antibiotic susceptibility tests in selected areas of southwest Ethiopian peoples regional state, Ethiopia. Vet Med (Auckl). (2023) 14:233–44. doi: 10.2147/VMRR.S435932, 38162823 PMC10757770

[27] Abdulkadir M Nigussie T Kebede IA. Isolation and identification of *Pasteurella multocida* and *Mannheimia haemolytica* from pneumonic small ruminants and their antibiotic susceptibility in Haramaya District, eastern Ethiopia. ScientificWorldJournal. (2024) 2024:5605552. doi: 10.1155/2024/5605552 PMC11039012 38655561

[28] Abdul-Ratha HA Najim TM Sadeq YK. Isolation and identification of *Pasteurella multocida* and *Mannheimia haemolytica* and the antimicrobial effect of *Plantago lanceolata* extract. J Anim Health Prod. (2025) 13:607–15. doi: 10.17582/journal.jahp/2025/13.s1.607.615

[29] Arnal JL Fernandez A Vela AI Sanz C Fernandez-Garyzabal JF Cid D. Capsular type diversity of *Mannheimia haemolytica* determined by multiplex real-time PCR and indirect hemagglutination in clinical isolates from cattle, sheep, and goats in Spain. Vet Microbiol. (2021) 258:109121. doi: 10.1016/j.vetmic.2021.109121, 34020174

[30] Townsend KM Frost AJ Lee CW Papadimitriou JM Dawkins HJS. Development of PCR assays for species- and type-specific identification of *Pasteurella multocida* isolates. J Clin Microbiol. (1998) 36:1096–100. doi: 10.1128/JCM.36.4.1096-1100.1998, 9542944 PMC104696

[31] Razin S Yogev D Naot Y. Molecular biology and pathogenicity of mycoplasmas. Microbiol Mol Biol Rev. (1998) 62:1094–156. doi: 10.1128/MMBR.62.4.1094-1156.1998, 9841667 PMC98941

[32] Xu X Yang F Zhang Q Xu Y Huang J Fu M . Development of a multiplex TaqMan qPCR assay for simultaneous detection and differentiation of four DNA and RNA viruses from clinical samples of sheep and goats. J Virol Methods. (2019) 266:58–64. doi: 10.1016/j.jviromet.2019.01.015, 30703412

[33] Nguyen TTT Nguyen VP Nguyen HT Nguyen TO Truong PH Ho TXT . Development of a multiplex PCR method for detection of *Pasteurella multocida* and *Mannheimia haemolytica*. Hue Univ J Sci Nat Sci. (2023) 132:69–79. doi: 10.26459/hueunijns.v132i1C.7165

[34] Kgotlele T Chota A Chubwa CC Nyasebwa O Lyimo B Torsson E . Detection of peste des petits ruminants and concurrent secondary diseases in sheep and goats in Ngorongoro district, Tanzania. Comp Clin Pathol. (2019) 28:755–9. doi: 10.1007/s00580-018-2848-5

[35] Zhao Z Peng J Lu Q Yuan L Tian M Jia S . Development and preliminary evaluation of multiplex PCR assays for the detection of bacterial respiratory pathogens in bovine and ovine. Vet Anim Sci. (2026) 34:100734. doi: 10.1016/j.vas.2026.100734, 42328207 PMC13277636

[36] Yang F Dao X Rodriguez-Palacios A Feng X Tang C Yang X . A real-time PCR for detection and quantification of *Mycoplasma ovipneumoniae*. J Vet Med Sci. (2014) 76:1631–4. doi: 10.1292/jvms.14-0094, 25649947 PMC4300380

[37] Bustin SA Benes V Garson JA Hellemans J Huggett J Kubista M . The MIQE guidelines: minimum information for publication of quantitative real-time PCR experiments. Clin Chem. (2009) 55:611–22. doi: 10.1373/clinchem.2008.112797, 19246619

[38] Thieulent CJ Carossino M Peak L Strother K Wolfson W Balasuriya UBR. Development and validation of a panel of one-step four-plex qPCR/RT-qPCR assays for simultaneous detection of SARS-CoV-2 and other pathogens associated with canine infectious respiratory disease complex. Viruses. (2023) 15:1881. doi: 10.3390/v15091881, 37766287 PMC10535912

[39] Hao F Tao C Xiao R Huang Y Yuan W Wang Z . Development of a multiplex real-time PCR assay for the detection of eight pathogens associated with bovine respiratory disease complex from clinical samples. Microorganisms. (2025) 13:1629. doi: 10.3390/microorganisms13071629, 40732137 PMC12300140

[40] Dutta E Loy JD Deal CA Wynn EL Clawson ML Clarke J . Development of a multiplex real-time PCR assay for predicting macrolide and tetracycline resistance associated with bacterial pathogens of bovine respiratory disease. Pathogens. (2021) 10:64. doi: 10.3390/pathogens10010064, 33450871 PMC7828349

[41] Zhang J Wang W Yang M Lin J Xue F Zhu Y . Development of a one-step multiplex real-time PCR assay for the detection of viral pathogens associated with the bovine respiratory disease complex. Front Vet Sci. (2022) 9:825257. doi: 10.3389/fvets.2022.825257, 35155658 PMC8825873

[42] Goto Y Fukunari K Suzuki T. Multiplex RT-qPCR application in early detection of bovine respiratory disease in healthy calves. Viruses. (2023) 15:669. doi: 10.3390/v15030669, 36992378 PMC10057971

[43] Buczinski S Broes A Savard C. Frequency of bovine respiratory disease complex bacterial and viral agents using multiplex real-time qPCR in Quebec, Canada, from 2019 to 2023. Vet Sci. (2024) 11:631. doi: 10.3390/vetsci11120631, 39728971 PMC11680374

[44] Fergusson M Maley M Geraghty T Palarea-Albaladejo J Mason C Rocchi MS. Validation of a multiplex-tandem RT-PCR for the detection of bovine respiratory disease complex using Scottish bovine lung samples. Vet J. (2024) 303:106058. doi: 10.1016/j.tvjl.2023.106058 38103886

