# Supplementary material for: Development and validation of a multiplex TaqMan real-time quantitative PCR assay for the simultaneous detection of six major bacterial respiratory pathogens in goats
Source: Front Vet Sci. 2026 Sep 17;13:1925484. doi: 10.3389/fvets.2026.1925484 (PMC13626985; doi:10.3389/fvets.2026.1925484)
Supplement: Supplementary file 1 [file Supplementary_file_1.DOCX]

**Supplementary Materials**

Development and Validation of a Multiplex TaqMan Real-Time Quantitative PCR Assay for the Simultaneous Detection of Six Major Bacterial Respiratory Pathogens in Goats

**Figure S1.** High-copy plasmid interference evaluation of Panel I. The three standard plasmids were normalized to the same concentration and tested individually or as an equal-copy mixture using the same per-target input. (A–C) Single-template amplification of Mo, Mmc, and Mccp, respectively. (D) Simultaneous amplification of the equal-copy mixture of Mo, Mmc, and Mccp. All three target channels remained detectable in the mixed-template reaction, with no target dropout under the tested high-copy conditions.

**Figure S2.** High-copy plasmid interference evaluation of Panel II. The three standard plasmids were normalized to the same concentration and tested individually or as an equal-copy mixture using the same per-target input. (A–C) Single-template amplification of Pm, Mh, and Sz, respectively. (D) Simultaneous amplification of the equal-copy mixture of Pm, Mh, and Sz. All three target channels remained detectable in the mixed-template reaction, with no target dropout under the tested high-copy conditions.

**Figure S3.** Expanded analytical specificity evaluation of the multiplex qPCR assay. (A) Panel I targets (1. Mo, 2. Mmc, 3. Mccp) and non-target pathogens 4–9. (B) Panel II targets (1. Pm, 2. Mh, 3. Sz) and the same non-target panel. Non-target pathogens were numbered as follows: 4. Mycoplasma bovis; 5. Escherichia coli; 6. bovine viral diarrhea virus (BVDV); 7. Mycoplasma arginini; 8. Trueperella pyogenes; and 9. bovine herpesvirus 1/infectious bovine rhinotracheitis virus (BoHV-1/IBRV). No target-specific amplification was detected for any of the six non-target pathogens.
