# Supplementary material for: Development and validation of a multiplex TaqMan real-time quantitative PCR assay for the simultaneous detection of six major bacterial respiratory pathogens in goats
Source: Front Vet Sci. 2026 Sep 17;13:1925484. doi: 10.3389/fvets.2026.1925484 (PMC13626985; doi:10.3389/fvets.2026.1925484)
Supplement: Supplementary file 2 [file Supplementary_file_2.DOCX]

**Supplementary Table S1. Agreement between multiplex qPCR and routine PCR for detecting six respiratory pathogens in 200 clinical samples**

| **Pathogen** | **qPCR+/PCR+** | **qPCR+/PCR-** | **qPCR-/PCR+** | **qPCR-/PCR-** | **Overall agreement (%)** | **Cohen's kappa** | **Exact McNemar P value** |
| --- | --- | --- | --- | --- | --- | --- | --- |
| Mo | 37 | 12 | 0 | 151 | 94.0 | 0.823 | <0.001 |
| Mmc | 40 | 3 | 0 | 157 | 98.5 | 0.954 | 0.250 |
| Mccp | 25 | 1 | 0 | 174 | 99.5 | 0.978 | 1.000 |
| Pm | 16 | 10 | 0 | 174 | 95.0 | 0.736 | 0.002 |
| Mh | 15 | 6 | 0 | 179 | 97.0 | 0.817 | 0.031 |
| Sz | 22 | 4 | 0 | 174 | 98.0 | 0.905 | 0.125 |

Abbreviations: Mo, Mycoplasma ovipneumoniae; Mmc, Mycoplasma mycoides subsp. capri; Mccp, Mycoplasma capricolum subsp. capripneumoniae; Pm, Pasteurella multocida; Mh, Mannheimia haemolytica; Sz, Streptococcus equi subsp. zooepidemicus.
