# Supplementary material for: Development and validation of a multiplex TaqMan real-time quantitative PCR assay for the simultaneous detection of six major bacterial respiratory pathogens in goats
Source: Front Vet Sci. 2026 Sep 17;13:1925484. doi: 10.3389/fvets.2026.1925484 (PMC13626985; doi:10.3389/fvets.2026.1925484)
Supplement: Supplementary file 3 [file Supplementary_file_5.DOCX]

**Supplementary Figure S4**

**Supplementary Figure S4.** Preliminary optimization of primer concentrations in the six uniplex qPCR assays. Equal final concentrations of the forward and reverse primers were evaluated at 0.10, 0.20, 0.30, and 0.40 µM, while the probe concentration, plasmid template input, reaction volume, and cycling conditions were kept constant. Panels A–F represent Mo, Mmc, Mccp, Pm, Mh, and Sz, respectively. For Mo, Mmc, Pm, and Mh, the two earliest and partially overlapping curves correspond to 0.30 and 0.40 µM; because increasing the concentration to 0.40 µM produced no meaningful improvement, 0.30 µM was selected as the provisional concentration. For Mccp and Sz, all four tested concentrations produced similar amplification profiles, and the lowest concentration of 0.10 µM was selected. During subsequent triplex balancing, the primer concentrations for Pm and Mh were further refined to 0.15 µM, whereas the selected concentrations for the other targets were retained. Final target-specific concentrations are reported in Table 1.
