# Supplementary material for: Development and validation of a multiplex TaqMan real-time quantitative PCR assay for the simultaneous detection of six major bacterial respiratory pathogens in goats
Source: Front Vet Sci. 2026 Sep 17;13:1925484. doi: 10.3389/fvets.2026.1925484 (PMC13626985; doi:10.3389/fvets.2026.1925484)
Supplement: Supplementary file 4 [file Supplementary_file_6.DOCX]

**Supplementary Table S3. Summary of probe-concentration optimization for the two triplex qPCR panels**

| **Panel** | **Target** | **Probe concentration range evaluated** | **Final selected concentration** |
| --- | --- | --- | --- |
| I  I  I | Mo | 0.05–0.30 µM | 0.25 µM |
|  | Mmc | 0.05–0.30 µM | 0.30 µM |
|  | Mccp | 0.05–0.30 µM | 0.05 µM |
| II  II  II | Pm | 0.05–0.30 µM | 0.25 µM |
|  | Mh | 0.05–0.30 µM | 0.30 µM |
|  | Sz | 0.05–0.30 µM | 0.05 µM |
